# The tree species matters: Biodiversity and ecosystem service implications of replacing Scots pine production stands with Norway spruce

**DOI:** 10.1007/s13280-019-01259-x

**Published:** 2019-09-24

**Authors:** Adam Felton, Lisa Petersson, Oscar Nilsson, Johanna Witzell, Michelle Cleary, Annika M. Felton, Christer Björkman, Åsa Ode Sang, Mats Jonsell, Emma Holmström, Urban Nilsson, Jonas Rönnberg, Christer Kalén, Matts Lindbladh

**Affiliations:** 1 Southern Swedish Forest Research Centre, SLU, Box 49, Rörsjöv 1, 230 53 Alnarp, Sweden; 2 Southern Swedish Forest Research Centre, SLU, Box 49, Sundsvägen 3, 230 53 Alnarp, Sweden; 3 Department of Ecology, SLU, Box 7044, 750 07 Uppsala, Sweden; 4 Department of Landscape Architecture, Planning and Management, Box 66, 23053 Alnarp, Sweden; 5 National Forest Agency, Bryggargatan 19-21, 503 38 Borås, Sweden

**Keywords:** Biological diversity, Climate change, Ecosystem services, Planted forest, Sustainability

## Abstract

The choice of tree species used in production forests matters for biodiversity and ecosystem services. In Sweden, damage to young production forests by large browsing herbivores is helping to drive a development where sites traditionally regenerated with Scots pine (*Pinus sylvestris*) are instead being regenerated with Norway spruce (*Picea abies*). We provide a condensed synthesis of the available evidence regarding the likely resultant implications for forest biodiversity and ecosystem services from this change in tree species. Apart from some benefits (e.g. reduced stand-level browsing damage), we identified a range of negative outcomes for biodiversity, production, esthetic and recreational values, as well as increased stand vulnerability to storm, frost, and drought damage, and potentially higher risks of pest and pathogen outbreak. Our results are directly relevant to forest owners and policy-makers seeking information regarding the uncertainties, risks, and trade-offs likely to result from changing the tree species in production forests.

## Introduction

Production forests can provide a broad range of ecosystem services, including biomass for materials and energy, habitat for biodiversity, environments for recreation, and non-wood forest products. Alternatively, production forest lands can be managed primarily for wood biomass, using uniform and intensive approaches to silviculture (Duncker et al. 2012). A key example is the growing global reliance on planted forests, which now comprise 7% of global forest area (FAO 2015), and the majority of forest lands in some European countries (Forest Europe 2011). Whereas intensive production forestry provides large amounts of biomass per unit area, it can have adverse implications for biodiversity and limit the ecosystem services provided, especially if extensive areas are uniformly managed (Felton et al. 2016a).

In Sweden, the majority of productive forest land is used for wood production, whereas approximately 10% is either formally or voluntarily protected (SLU 2018). Swedish forestry is highly effective, enabling this high-latitude country with just 1% of the world’s forest area, to be the third largest exporter of pulp, paper and sawn timber (SFIF 2018). Sweden achieves this primarily by rotationally clear felling even-aged stands of either native Norway spruce (*Picea abies*) or native Scots pine (*Pinus sylvestris*), which together comprise 80% of Sweden’s standing volume (SLU 2018). Though successful in terms of biomass production and harvesting efficiency, the widespread uniformity of current silviculture has raised concerns regarding impacts on forest biodiversity, the breadth of ecosystem services provided, and forest resilience (Ulmanen et al. 2012). In this regard since the early 1990s, the Swedish Forest Act gives equal status to environmental and production objectives (Gov. bill 1992/93:226). A specific concern is the regeneration of what were traditionally Scots pine production stands with Norway spruce (SFA 2018a). As a result, Norway spruce is now the most commonly chosen tree species for regenerating sites in most southern Swedish regions, regardless of whether the site is of low, medium or high-soil fertility (SFA 2018c). A key motivator is that Norway spruce combines high-production volumes, good economy, well-established management regimes, and competitive growth rates (Ekö et al. 2008), with the additional benefit of being relatively unpalatable to browsing herbivores (Lodin et al. 2017; SFA 2017). In some regions, government-supported efforts are trying to avert this spiral of converting Scots pine stands to Norway spruce (SFA 2017), and parallel efforts to increase the availability of alternative forage in the landscape may further help avoid such developments (Bergqvist et al. 2018). The most recent assessments indicate however that the conversion of pine sites to spruce has only slowed down, but not stopped (SFA 2017).

It is within this context that we synthesize the potential implications for biodiversity and a range of cultural, provisioning, and regulating ecosystem services from the conversion of Scots pine stands to Norway spruce, focusing on southern Sweden (Götaland) where this practice is most prevalent. We use the ecosystem services framework to evaluate outcomes, which refers to the benefits people obtain either directly or indirectly from ecosystems (Nahlik et al. 2012). Our primary aim is to synthesize a diverse range of socio-ecological implications from changing from one dominant tree species to another in production forestry, and highlight the broad range of resulting biodiversity and ecosystem service implications.

### Synthesis methods

We summarized the current state of scientific evidence regarding the implications of converting Scots pine stands to Norway spruce for key elements of forest biodiversity (bryophytes, epiphytic lichens, saproxylic beetles, birds, large herbivores), and a range of forest-related cultural (recreation and esthetics), provisioning (biomass production, wood product diversity), and regulatory services (damage risk from storms, drought, fire, frost, pests, pathogens). Due to the range of issues addressed, and space constraints, our results are best seen as a condensed synthesis. Hence, supporting services such as soil productivity, regulation of water flow and quality, and climate regulation are not included in the assessment. The choice of topics was targeted towards issues frequently raised by forest stakeholders (e.g. production outcomes, damage risk, recreation, biodiversity) with respect to stand conversion (Lidskog and Sjödin 2014; Lodin et al. 2017; SFA 2017), and was also dictated by the expertise of participating researchers. As the topics chosen for inclusion, as well as the boundary delineation for each topic are to some extent subjective, our results cannot be used to summarize the entirety of potential costs and benefits derived from each stand type.

We searched for relevant published studies using electronic databases and different combinations of Boolean search terms. The databases used were Web of Science (http://www.isiwebofknowledge.com/), Google Scholar (http://scholar.google.com.se/), and Scopus (https://www.elsevier.com/solutions/scopus). For example, the following search-terms were used to find relevant studies on birds: (“Scots pine” OR “*Pinus sylvestris*” OR “Norway spruce” OR “*Picea abies*”) AND “bird*”. We also obtained papers from colleagues and through reference lists from published studies, including major review articles and books on managed production forests, government studies, and reports. We prioritized studies conducted within the Fenno-Scandinavian region, due to their increased silvicultural and bio-geographical relevance. We focused on stands designed specifically for the even-aged production of Norway spruce (hereafter spruce) or Scots pine (hereafter pine). We summarize tree and stand attributes associated with standard silvicultural practice in southern Sweden in Table 1. The spatial resolution of interest was the stand level, though we also discuss landscape-level implications.

**Table 1:** Summary of stand and eco-physiological attributes (for each tree species) associated with standard silvicultural practice of Norway spruce and Scot’s pine stands in southern Sweden

Tree and stand attributes	Norway spruce	Scots pine
Regional history	Immigrated 1000–2000 years ago. Became the most common 70–100 years ago	Immigrated > 10 000 year ago. Around 25% of all trees during most of the Holocene
Regeneration	Planting	Planting (occasional seed tree-aided natural regeneration or direct seeding)
Felling/stand structure	Rotational clearcutting of even-aged stands	Rotational clearcutting of even-aged stands
Rotation length (years) (rich site-poor site)	45–90	60–90
Target stem density per ha	550–1000	550–1000
Thinning regime	1–2 commercial thinnings	1–3 commercial thinnings
Potential lifespan (years)	300+	500+
Growth rate	Slow growth in young stands, high and sustained growth late in the rotation	Faster development in young stands but reduced at the end of the rotation
Soil types used	All fertility and soil moisture classes, except extremely poor or dry sites	Poor to intermediate fertility and dry sites
Root architecture	Plate root systems with sinker roots (more modified by soil conditions)	Tap root system (less modified by soil conditions)
Crown structure	Longer crown	Shorter/higher crown
Leaf area index	5–10	2–5
Needle structure	More clustered	Less clustered
Understory light levels	Lower	Higher
Bark crenulation	Low for all tree ages	High in older trees
Bark pH	More acidic	Less acidic

Where possible we provide quantitative results (e.g. in the production section). However, in general, and specifically in relation to Tables 1 and 2, we restrict ourselves to evaluating the general direction of change expected, rather than quantifying the magnitude of an effect. This is due to both limited knowledge regarding many effect sizes, and large variability across issues in terms of relevant metrics. Instead of the magnitude, we estimate the degree of confidence in the expected direction of change (Tables 1 and 2). Confidence levels were subjective scores that varied from “possible” to “highly probable” depending on the cumulative weight and consistency of available study findings. In cases where the impact of changing from pine to spruce on a particular issue is unlikely to extend beyond the normal variation associated with pine-dominated stands, we assessed such impacts as neutral.

**Table 2:** Summary of expected changes at the stand level for species diversity, community composition and red-listed species due to shifting from Scots pine to Norway spruce. Outcomes are graded as positive results “↑”, negative outcomes “↓”, neutral/no change outcomes “◯”, and uncertain outcomes ↕. Species diversity takes into consideration both species richness and abundance. The delta symbol “∆” indicates a change in community composition, a key consideration when determining landscape-scale biodiversity impacts. “Effect modifiers” indicates management that strongly impacts on outcomes. Confidence levels (i.e. *,**,***) represent “possible”, “probable”, and “highly probable” respectively, but are not relevant to “uncertain” outcomes

Biodiversity	Expected general change			Effect modifier
	Stand–level species diversity	Community composition	Red-listed species	
Vascular plants	↓**	∆***	↓*	Stand density / canopy cover
Bryophytes	↑*	∆***	↕	Rotation length, coarse woody debris retention
Epiphytic lichens	↕	∆***	↓*	Rotation length
Saproxylic beetles	↕	∆***	↕	Dead wood occurrence Stand insolation / temperature
Birds	↑*	∆***	◯**	Broadleaf retention levels, rotation length
Large herbivores	↓**	◯*	N/A	Stand density / canopy cover; broadleaf retention

## Results

### Implications for forest biodiversity

#### Vascular plants and bryophytes

Overstory effects on understory vascular plants is largely dictated by interspecific competition, mediated by soil and light conditions (Kuusipalo 1985a). The most distinct difference between pine and spruce stands is lower understory light levels beneath the later (see Table 1; Kuusipalo 1985b; Bäcklund et al. 2015). As a result, the field layer abundance of spruce stands is generally lower than in pine (Bäcklund et al. 2015) and declines rapidly as stem density increases (Hedwall et al. 2013; Tonteri et al. 2016). Understory light levels can be particularly low in southern Sweden due to high-stem densities of spruce, which may cause vascular plants to be replaced by bryophytes or patches lacking vegetation (Esseen et al. 1997).

In contrast, light is rarely limiting in pine stands, and understory composition is primarily determined by competition (Tonteri et al. 1990). In dry and high light conditions, ground-living species of lichens benefit (Okland 1995), but tend to decline with increased stand age and stem densities (Bäcklund et al. 2015). At later successional stages, competitive dwarf shrubs such as bilberry (*Vaccinium myrtillus*) and cowberry (*V. vitis*-*idaea*) can dominate and prevent other species from establishing (Tonteri et al. 1990), thereby lowering species richness (Widenfalk and Weslien 2009). However, dwarf shrubs provide food resources that benefit manyf taxa (see below).

In summary, shifting from pine to spruce can be expected to alter understory plant communities, with landscape scale implications due to a reduced prevalence of pine stands. However, we do not expect red-listed vascular plant species in either stand type (ArtDatabanken 2018). Although some red-listed species are favored by spruce coarse woody debris (Hallingbäck 1996), other requirements important for their occurrence, such as forest cover continuity, are generally missing.

#### Epiphytic lichens

Three key interacting factors dictate lichen species diversity in the production of forest stands, i) tree species’ substrate, ii) time available for colonization, and iii) microclimate. Lichen establishment and growth is strongly affected by host tree species’ substrate, which can vary in bark pH, structure, and stability (Johansson et al. 2007). The bark of pine differs from spruce as it is slightly less acidic and thus more hospitable to some lichen species (Marmor et al. 2010). Pine bark also thickens with age, and exfoliate over time; which benefits habitat variation, but can limit time for colonization (Kuusinen 1996). Rotation lengths are also a key determinant of lichen communities, as lichen biomass accumulates slowly over time (Dettki and Esseen 2003), and both species richness and the number of red-listed species increases with stand age in both stand types (Marmor et al. 2011; Bäcklund et al. 2016). In even-aged conifer stands, reduced light availability can be negative for photosynthesizing organisms, including lichens (Gauslaa et al. 2007). For this reason, decisions regarding rotation length and stem densities (Table 1) alter the tree sizes and bark conditions available for colonization, and the suitability of the microclimate (Roberge et al. 2016; Felton et al. 2017).

The results of studies contrasting epiphytic lichen species richness vary widely, as pine may support lower (Marmor et al. 2011; Bäcklund et al. 2016), approximately equal (Uliczka and Angelstam 1999), or higher species richness (Hyvärinen et al. 1992) than spruce. Assessments of community composition do however indicate that pine may support a higher richness of photophilic species (Bäcklund et al. 2016), and in some studies, a higher abundance of foliose lichens (Uliczka and Angelstam 1999) than spruce. Whereas spruce may provide habitat for a higher abundance of fructicose lichens (Uliczka and Angelstam 1999). Observed differences in the lichen communities supported, and general limits to lichen dispersal, suggest that lichen diversity may be adversely affected by converting pine sites to spruce at landscape scales.

#### Saproxylic beetles

Saproxylic beetles inhabit dead wood in various states of decay, whereby the tree species, dead wood diameter, decay class, and insolation levels are important determinants of their community composition and diversity (Stokland et al. 2012). There are an estimated 360 species of saproxylic beetles using spruce in Sweden, relative to the 300 that use pine (Stokland et al. 2012). The larger number of species associated with spruce is reflected in assessments of species richness conducted on stumps in Sweden (Jonsell and Hansson 2011), logs in Germany (Gossner et al. 2016) and dead trees in Poland (Hilszczanski et al. 2016). The community composition supported also differs. Whereas a high proportion of saproxylic species can use both tree species in Sweden (232 species), a substantial number rely exclusively on either pine (68 species) or spruce (128 species) (Dahlberg and Stokland 2004). This difference between tree species is largest when the dead wood is fresh (Jonsell et al. 1998), as in later stages species occurrence is determined more by the developing fungal flora (Jonsell et al. 2005; Stokland 2012). Both species diversity and community composition can also differ due to higher insolation levels beneath pine production stands than spruce stands (Kuusipalo 1985b).

In terms of threatened taxa, there are slightly more red-listed saproxylic beetle species associated with spruce than pine (Jonsell et al. 1998). However, productions forests usually lack the wood habitats associated with natural forests and natural disturbance processes. Therefore, the probability that red-listed species will occur is low, unless source populations are close by (Similä et al. 2003). In summary, spruce can support a higher number of saproxylic beetle species than pine, but the higher levels of insolation provided by pine will favor the many species benefiting from warmer microclimates. In either regard, substantial differences in community composition are expected. The effects of tree species change on saproxylic beetles will largely depend on management regimes and the amount and type of dead wood retained.

#### Bird communities

Although the bird communities of pine and spruce production stands are likely to consist of conifer-associated generalists, some differences in species composition and diversity can be expected. For example, compared to spruce, pine forests can support exclusively, or host larger populations of tree pipit (*Anthus trivialis*), redstart (*Phoenicurus phoenicurus*), and spotted flycatcher (*Muscicapa striata*) (Gjerde and Saetersdal 1997). This may stem from the more open canopy conditions of pine favouring the foraging requirements of insectivores (Edenius 2011). In addition, differences between common crossbill (*Loxia curvirostra*) and parrot crossbill (*Loxia pytyopsittacus*) in their consumption of spruce versus pine cones (Marquiss and Rae 2002), suggests likely differences in stand use. Turnover may also be expected among tit species. For example, crested tit (*Lophophanes cristatus*) is often associated with pine (Gjerde and Saetersdal 1997), whereas coal tit (*Periparus ater*) is more associated with spruce (Haapanen 1965). Goldcrest (*Regulus regulus*) may also contribute to differences in community composition, as this species is often associated with spruce (Haapanen 1965). Targeted surveys of 55 and 80-year-old spruce and pine production stands in southern Sweden found that bird community composition differed in line with these expectations, but that older spruce stands can support higher bird diversity than pine, with effects depending on rotation length and broadleaf retention practices (Lindbladh et al. 2019).

Spruce and pine stands can thus be expected to differ in bird community composition, and species diversity may be higher in spruce stands in some contexts (Table 2). Concerns may thereby be raised regarding the landscape-level implications for bird communities from converting pine stands to spruce, and the shortening of rotation lengths in spruce stands.

#### Large herbivores

Sweden has comparatively high-population densities of large herbivores (Angelstam et al. 2017), including e.g. moose (*Alces alces*) and roe deer (*Capreolus capreolus*). A primary determinant of their population density, besides hunting, is the spatial and temporal availability of adequate browse, which includes the foliage, twigs, and bark of trees and understory shrubs. Whereas spruce is sometimes browsed, pine and broadleaf trees are generally preferred (Månsson et al. 2007). In young spruce stands, naturally regenerating broadleaved trees are often browsed (Wam et al. 2010); whereas in young pine stands, the production stems are also selected by the browsers. As stands gets older, forage availability is primarily determined by the understory vegetation that develops and the extent to which naturally regenerating trees are retained. Large herbivores also feed extensively on dwarf shrubs (e.g., Cederlund et al. 1980), the abundance of which can be reduced in the low light availability of denser production stands (Hedwall and Brunet 2016; SLU 2017).

In summary, a shift from pine to spruce stands in southern Sweden, and an associated decline in the cover of shade-intolerant broadleaves and dwarf shrubs (Hedwall et al. 2013; Hedwall and Brunet 2016) will thus likely reduce the availability, and alter the spatial distribution, of food resources for large herbivores. The resultant spatial and temporal concentration of food will likely intensify competition among deer species, with negative repercussions for those herbivores most dependent on forest foods. However, we do not expect community composition to change at the stand level.

## Cultural services

### Esthetics and recreation

In visual preferences studies, pine stands generally receive higher preference and scenic beauty scores than spruce (Brown and Daniel 1986; Tyrväinen et al. 2003). Non-image based studies also find a preference for pine when respondents specify which stand type they prefer in a forest landscape (e.g. Mattsson and Li 1994). Abundant undergrowth can, however, cause pine stands to be rated similarly to spruce in esthetic value (Tyrväinen et al. 2003). In addition, people’s preference generally increases with tree size and stands at later developmental stages (Silvennoinen et al. 2001; but see Gundersen and Frivold 2008). Preference can be particularly strong for stands possessing large pine trees (e.g. Brown and Daniel 1986), and modeling studies link improved scenic beauty with pines over 10 m tall (Silvennoinen et al. 2001). The higher esthetic scores provided to pine stands is also likely to be influenced by people’s preferences for recreational environments, as scenic beauty scores are strongly correlated to recreational evaluations (Daniel et al. 1989). In terms of restorative benefits, Sonntag-Öström et al. (2011) found that pine forests were preferred over spruce for visitation by individuals recovering from stress. However, context matters, and near urban environments spruce may have higher restorative effects if their density insulates individuals from the urban matrix (Hauru et al. 2012).

In summary, a consistent shift away from pine to spruce stands is likely to decrease the esthetic value of production forest landscapes. The recreational value of production forest lands may also be affected, if a shift away from pine limits the prevalence of dwarf shrubs and the opportunity provided for picking berries (see above). Likewise, if a higher prevalence of spruce stands reduces food resources for large herbivores and birds, this may reduce the recreational benefits derived from larger populations of game species.

## Provisioning services

### Production outcomes

When evaluating forestry production, it is important to consider the tree species’ preferred site conditions and growth characteristics. In Sweden, both tree species are accepted by forest law as crop trees on all fertility and soil moisture classes, with the exception that spruce is not used on extremely poor sites in northern Sweden (Swedish Forest Agency 2017). Pine is, however, recommended for poor to intermediately fertile dry sites, whereas spruce is recommended for intermediate to fertile mesic to moist sites (Albrektson et al. 2012). Their growth characteristics also differ, as pine grows more rapidly than spruce at younger ages, whereas spruce has a more sustained growth over time (Table 1). For this reason, comparison of production outcomes is difficult when comparing stands of young age and should instead include whole-rotation analysis involving the culmination of mean annual increment (MAI).

There are three main sources of data used to compare tree species growth and related production outcomes. The first uses survey data from proximate stands with similar site conditions. Studies using this method, and paired sites across Sweden (Leijon 1979) and Norway (Öyen and Tveite 1998), indicate that the growth rates of pine is inferior to spruce on most sites, with the exception of low fertile and dry sites. Alternatively, growth performance can be inferred using thousands of national forest inventory plots with species-specific site indices (e.g. using site properties like field vegetation, see Hägglund and Lundmark 1977). Using this method, pine produces on average 70% of the biomass of spruce, with production differences decreasing with increasing latitude (Ekö et al. 2008). However, these results require caveats because; (i) silvicultural treatment history and management intent is unknown, (ii) site and tree species selection are non-independent, (iii) differences in management guidelines can skew comparisons (e.g. over-thinning of pine can limit production), and (iv) errors occur in site index conversion between tree species.

These limitations can, however, be overcome using controlled experiments assessing long-term tree species production outcomes at the same site. However, such trials of spruce and pine are rare in Sweden (Nilsson et al. 2012). One controlled experiment from northern Sweden found that pine produced more volume on all but the most fertile sites; with the total gross volume of spruce approximately 30% of pine (Nilsson et al. 2012). An additional pairwise comparison of volume growth on a medium fertile site in central Sweden (one site), found that at 57 years of age, the volume growth of pine outperformed spruce by over 100% (Holmström et al. 2018). Furthermore, Drössler et al. (2018) found that pine provided 107% of spruce periodic annual increment on three fertile sites (SI 30-36 for pine) in southern Sweden; highlighting Scots pine’s potential as a viable alternative even on fertile sites. However, unpublished results from young tree species experiments on fertile sites in southwestern Sweden indicate that growth of Norway spruce is significantly higher than for Scots pine. In summary, the few available experimental comparisons that exist in southern Sweden indicate that growth of pine is superior to spruce on low to medium fertile sites, and that the production capacity of pine may be consistent with spruce, except on the most fertile sites.

### Wood products/quality provided

Despite an overlap in the type of wood products sourced from spruce and pine, there are differences in end products, quality classes, and market prices. Whereas spruce wood is often used in construction and packaging, pine provides a wider range of sawn timber categories and quality classes, and is more generally used for planed wood (Swedish Forest Agency 2014), and fine carpentry end products (Saarman 1992). Note, however, that whereas there is a price/quality relationship, and high quality pine-timber is priced higher than spruce timber, spruce wood is generally priced higher than pine (SDC 2015; Södra 2018a, b), and provides 50% of the round wood used in the Swedish forest industry compared to pine’s 40% (SDC 2017). Spruce and pine are also extensively used in the pulp and paper industry. There are two main assortments: non-specific conifer, and spruce pulpwood, the latter returning slightly higher prices (Nylinder and Fryk 2015). In summary, current market prices appear to favor spruce biomass in general, and the forest industry is adapted to processing this tree species. Nevertheless, spruce does not provide the same range of wood products as pine.

## Regulatory services

### Climate suitability and abiotic risks

Under moderate to high greenhouse gas emission scenarios, some projections indicate growing condition improvements for both spruce and pine, though pine is projected to experience a higher relative increase in net primary production (Bergh et al. 2010). However, climate change is also associated with altered disturbance regimes (Grundmann et al. 2011), including to storms, droughts, fires, and pest or pathogen outbreaks, which likewise alter the growth and mortality rates of tree species. These kinds of events are especially hard to predict, but their associated risks are projected to increase over coming decades in many regions of Europe (Seidl et al. 2014). Notably, some projections indicate drier summers in southern Sweden, which can be expected to favor pine relative to spruce (Eriksson et al. 2015).

#### Drought damage

The risk of drought damage and mortality is of particular concern to spruce, especially when grown in warmer and drier conditions (Spiecker 2000). Thiele et al. (2017) recommends that sites with low soil–water holding capacity should be avoided when growing spruce. Pine is less vulnerable to drought (Klein 2014), and is thus better adapted to warmer and drier summers and the water stress that comes with increasing size (Zang et al. 2012). Warmer and drier conditions are projected to increase over the coming century in many areas of Sweden, where mean annual temperatures may increase by 3–7 °C with associated increases in the frequency of heatwaves and droughts (Kjellström et al. 2014). Converting pine to spruce under such conditions will thus increase the risk of drought damage.

#### Storm damage

Whereas climate projections do not provide clear indications of altered frequencies or intensities of storms in Sweden (Kjellström et al. 2014), windthrow increases with wet, mild winters with less soil freezing. Over the last century storm damage has increased in Sweden (Nilsson et al. 2004), largely due to the increased use of spruce monocultures (Schlyter et al. 2006). Because spruce is vulnerable to storm damage (Valinger and Fridman 2011), the increased use of spruce may increase storm damage risks (Blennow et al. 2010), as has occurred in Germany (Griess et al. 2012) and Finland (Suvanto et al. 2016). Storm risks in spruce stands can be reduced by shortening rotation lengths, but this adaptation measure has associated impacts on biodiversity and ecosystem services (Roberge et al. 2016; Felton et al. 2017).

#### Fire damage

Beyond external factors, the vulnerability of a stand to fire depends on fuel availability, its vertical distribution, and flammability (Fernandes et al. 2008; Schelhaas et al. 2010). Overall, pine is more fire-adapted than spruce (Engelmark et al. 1994), due to its relatively thick bark and higher crown (Angelstam and Kuuluvainen 2004), which reduces the risk of crown fires (Agee and Skinner 2005). However, in spruce stands there is usually a lower risk of fire ignition due to moister microclimates and reduced ground vegetation. As a result, possible ignition days each year are three to four times higher in pine than spruce stands (Tanskanen et al. 2005). In summary, the risk of fire is generally higher in pine stands, but these stands are also better fire-adapted. Large uncertainties remain especially regarding the fire risk for spruce on drier soil types, especially during drought years. Under such conditions, fires may be stand replacing for spruce, but non-stand replacing for pine.

#### Frost damage

When spruce shoots flush, they are particularly vulnerable to frost-induced fatality. Comparatively, pine is fairly tolerant to early and late season frosts, though tolerance levels vary with provenance (Govindarajulu 2014). As a result, frost damage on pine is almost non-existent in southern Sweden, whereas, the use of late flushing provenances, bare-rooted seedlings, and soil scarification may be needed to reduce frost damage risks in spruce seedlings (Langvall et al. 2001).

### Biotic risks

#### Insect pests and pathogens

A small number of insect species are responsible for the majority of pest damage to Swedish forests. Of greatest concern is the Eurasian spruce bark beetle (*Ips typographus*) which feeds primarily on weakened or freshly wind-thrown spruce trees. Bark beetles are also vectors for blue-staining ascomycete fungi (e.g. *Endoconidiophora polonica*) (Wingfield et al. 1993), which further degrades wood quality (Kuroda 2005). Increasing the stem density and prevalence of mature spruce stands is likely to raise the potential for bark beetle outbreaks (Overbeck and Schmidt 2012). The risk of mortality may further increase if a tree’s defensive capacity is limited by poor soil conditions or drought (Komonen et al. 2011). Storm damage can also increase the risk of outbreak due to associated increases in breeding habitat (Potterf and Bone 2017). Other bark beetle species, such as *Pityogenes chalcographus* and *Polygraphus poligraphus* that currently rarely cause severe damage (Eidmann 1992), have the potential to increase their pest severity if the intensity and frequency of stressed trees increases. The pine weevil *Hylobius abietis*, is the other major pest species in Sweden. However, we cannot infer that the risk of damage will alter due to this change in tree species. The planting of spruce on pine sites can however raise concerns regarding the potential for colonization and outbreak by pests normally associated with other tree species (Dalin and Björkman 2006).

In terms of pathogens, *Heterobasidion* spp. is of primary concern, both in spruce and pine. It infects freshly cut stumps or wounds and spreads through connecting root systems, causing extensive heartwood decay (Woodward et al. 1998). The resultant impacts on tree growth, windthrow risk, and degraded wood products (Bendz-Hellgren et al. 1998), are estimated to cause 1 billion SEK in economic losses per year in Sweden. The two species known to exist in Sweden, *H. parviporum* and *H. annosum* (Korhonen et al. 1998), mainly infect spruce, but can also infect pine (Korhonen 1978). However, susceptibility studies in seedlings, as well as growth rate studies in wood, clearly show higher risks for spruce (Zaļuma et al. 2016). On average, 25% of mature spruce trees at final felling are infected by root rot (Thor et al. 2005), and approximately 75% is due to *Heterobasidion* (Stenlid 1987). Notably, the risk for infection by *H. annosum* is higher on certain soil types, including well-drained sandy and shallow soils (Huse 1983). Thus, the planting of spruce on pine soil types may result in maladaptation of the host tree and a higher risk of infection. The canker-causing fungus *Neonectria fuckeliana* is also becoming an increasing problem for spruce (Pettersson et al. 2018), though it is unknown whether elevated risks will occur for spruce in this context.

Pathogen risks to pine also require consideration, though these risks do not appear to motivate conversion to spruce in Sweden. For example, epidemics of the *Gremmeniella abietina*-causing Scleroderris canker are of potential concern in pine (Wulff et al. 2006), as infection can reduce tree growth (Wang et al. 2017). More generally, a key concern in southern Sweden is the potential risk of new diseases, such as needle and tip blights that may become established due to changes in climate or forest management.

To summarize, whereas both tree species are susceptible to a spectrum of pests and pathogens, the planting of spruce on sites more suitable for pine may cause physiological stress that increases its vulnerability to damage by insect pests and pathogens. Furthermore, as higher density and abundance of susceptible host trees can increase the incidence and severity of damage caused by pests or pathogens (Prospero and Cleary 2017), the additional prevalence of spruce stands, and the trend towards denser planting, may further elevate risks.

#### Large browsing herbivores

High browsing pressure is especially damaging to young regenerating stands of pine (Wallgren et al. 2013). For example, recent assessments find that moose damage occurs on 12–20% of young pine stems (average 16%) among southern Swedish counties (SFA 2018c), well in excess of the 5% damage levels deemed acceptable by the forest industry (SFA 2017). As a result of the reduced risks of browsing damage in spruce stands, browsing damage is a strong motivation for the continued or expanded establishment of spruce on traditional pine sites (Lidskog and Sjödin 2014; Lodin et al. 2017). Note, however, that sustaining large herbivore population levels in combination with the increased establishment of spruce is likely to increase browsing pressure on remaining stands of pine (Wallgren et al. 2013; Bergqvist et al. 2014).

## Discussion

Our analysis revealed a complex matrix of outcomes for forest biodiversity and ecosystem services arising from the conversion of existing production pine stands to spruce. The majority of these results were negative, and stemmed primarily from three related and often interacting sources; (i) risks specific to spruce, (ii) risks associated with spruce establishment on less-suitable sites, and (iii) the resultant increased uniformity of production forest lands. First, the evidence available indicates that the use of spruce as a production tree species instead of pine increases stand vulnerability due to damage from storms, drought, frost, Eurasian spruce bark beetle, and *Heterobasidion* spp. root and butt rot (Table 3). Second, these risks are likely to be worsened if spruce trees experience physiological stress due to their establishment on sites better suited to pine. Not only can poor site conditions limit growth and production in spruce, but may also weaken the trees’ capacity to cope with the abiotic and biotic disturbances highlighted. The primary concern is that this could result in a ‘decline spiral’, stemming from the interaction of ‘inciting’, ‘predisposing’, and ‘contributing’ factors (Manion 1991; Allen et al. 2010). For example, an abiotic stress such as drought has the potential to incite the eventual mortality of trees that are already under stress due to predisposing factors such as poor site conditions. Under these conditions trees may then finally succumb due to additional contributing factors such as stem and root damage by insect pests and fungal pathogens (Manion 1991; Allen et al. 2010). More studies are needed to quantify such risks.

**Table 3:** Expected implications at the stand level of pine conversion to spruce for ecosystem services. Outcomes are graded in terms of positive outcomes “↑”, negative outcomes “↓”, and uncertain outcomes ↕. “Effect modifier” indicates management that has a strong impact on outcomes. Confidence levels (i.e. *,**,***) represent “possible”, “probable”, and “highly probable” outcomes, but are not relevant to “uncertain” outcomes

Ecosystem services	Positive or negative outcomes	Effect modifiers
*Provisioning*		
Biomass production	↓*	Varies with type and extent of disturbance (e.g. browsing pressure vs. storm damage)
Product diversity	↓*	
Wood prices	↕	
*Cultural*		
Forest aesthetics	↓**	
Hiking	↓**	
Hunting	↓*	
Bilberry picking	↓***	
Stress recovery	↓**	Potential to improve in urban areas
*Regulatory services*		
Abiotic risks		
Projected outcomes due to:		
Climate damage	↓**	Extent of future GHG emissions
Storm damage	↓***	When thinning and harvest takes place
Drought damage	↓***	
Fire damage	↕	Ignition risk may be lower in spruce, but damage higher if a fire occurs; unknown implications of spruce on dry sites
Frost damage	↓***	
Biotic risks		
Projected outcomes due to:		
Browsing damage	↑***	However, conversion may increase / focus landscape scale damage
Spruce bark beetle damage	↓***	
Other bark beetle damage	↓**	Tree stress may allow other bark beetles to become pest species
Root rot damage	↓***	Higher spruce stem densities are likely to increase risks

Third, these concerns are compounded by the fact that spruce is already the most common tree species in the region, and continued expansion onto pine sites will make production forest lands more uniform in both tree composition and silvicultural practice (e.g. regeneration practices, rotation lengths). Relying extensively on any single approach to production forestry is inconsistent with government efforts to diversify production forestry (SFA 2018b), societal expectations that production forests should provide a diverse range of goods and services (Gustafsson et al. 2012), and recommended strategies for mitigating the risks and uncertainties of anthropogenic climate change and associated abiotic and biotic disturbance (Seidl 2014). Furthermore, differences between pine and spruce stands in the habitats provided, environments created, and species supported (Table 2), means that long-term costs to at least some aspects of landscape-scale biodiversity can be expected if these conversions continue. As these changes are taking place in the most densely populated region of Sweden, greater societal impacts can also be expected from the resultant reductions in recreational and esthetic forest values and the provision of non-wood forest products (see Table 3).

If our results were encompassing, and the choice of tree species was simply a case of weighing these results equally, then the retention of pine stands appears clearly preferable to spruce conversion. However, our results do not and cannot address the entirety of concerns relevant to forest owners and other stakeholders. At best our results capture an important subset of the complex suite of incentives and disincentives for choosing a production tree species. Furthermore, forest owners vary in how they prioritize concerns, uncertainties, and benefits (Ingemarson et al. 2006; Puettmann et al. 2015). Many forest owners prioritize the need to limit browsing damage in young stands (Lidskog and Sjödin 2014; Lodin et al. 2017), which was one of the few stand-level benefits identified with converting to spruce. Stand regeneration is a primary cost associated with production forestry in Sweden, as over 80% of Swedish forest owners regenerate with commercially improved seedlings. Browsing damage not only risks this investment, but can also compromise the stand’s subsequent development and production outcomes (Nilsson et al. 2016).

The importance to forest owners of avoiding browsing damage was revealed by a large storm that struck southern Sweden in 2005, felling the equivalent of a year’s national harvest of primarily spruce-dominated stands (Svensson et al. 2011). Despite the demonstrated vulnerability of spruce to such storms (Valinger and Fridman 2011), and compensatory governmental funding to encourage regeneration with broadleaf alternatives (Wallstedt 2013), forest owners still used spruce for 90% of the replanted area (Valinger et al. 2014). Subsequent evaluations determined that many forest owners avoided using alternatives to spruce specifically because of the vulnerability of these other tree species to browsing damage (Lidskog and Sjödin 2014; Lodin et al. 2017). In addition, forest owners questioned the rationale of converting from spruce to alternative production tree species with unknown suitability to site conditions (Lidskog and Sjödin 2014). Despite the fact that similar concerns could be raised regarding spruce’s suitability to pine sites in the circumstances we assess (see Tables 1, 2), this has not prevented the widespread conversion to spruce. These results indicate either a lack of awareness or appreciation for the risks identified (Tables 2, 3), and/or the overriding impact that browsing damage aversion has on forest owners and managers.

The circumstances and types of dilemmas faced by forest owners and policy-makers outlined in this study are unlikely to be limited to Sweden. An increasing proportion of the world’s forest area is intensively managed for wood production (Warman 2014; Payn et al. 2015), often resulting in relatively uniform, even-aged and simplified forest states. If such management practices are applied to extensive areas, adverse implications can be expected for biodiversity conservation, the breadth of ecosystem services provided, and system resilience (Lindenmayer and Franklin 2002). Furthermore, the large-scale adoption of any single approach to natural resource management has the potential to become self-reinforcing, with future-expanded use driven to some extent by path-dependencies (Mahoney 2000; Boonstra and de Boer 2014). This can result in a system entering a so-called ‘social-ecological trap’ (sensu Steneck 2009), in which feedbacks between the social and ecological aspects of the system lead toward an undesirable state that may be difficult or impossible to reverse (Cinner 2011). As seen in this study, human modification of the landscape, and competing goals for forest and game, has resulted in levels of browsing pressure that constrain the choice of production tree species. Furthermore, the widespread ongoing conversion of pine to spruce has the potential to increase browsing activity and damage in remaining stands of pine, and thereby perpetuate the spiral of stand conversion (Wallgren et al. 2013; Bergqvist et al. 2014). Meanwhile, anthropogenic climate change compounds uncertainties when projecting the future returns and risks associated with tree species choices (Petr et al. 2014; Torssonen et al. 2015). These circumstances can cause forest owners to focus during stand establishment on immediate tangible concerns (e.g. mitigating browsing damage), at the expense of less tangible future potential risks (e.g. storm damage) (Lidskog and Sjödin 2014), and longer term social-ecological impacts and uncertainties. Halting rather than merely slowing the undesirable cumulative effects of such a ‘tyranny of small decisions’ (sensu Kahn 1966) will presumably require extensive and prolonged government-coordinated efforts. In this regard, we can only speculate as to whether a substantial reduction in browsing pressure alone would be sufficient to reduce the current preference for planting spruce (see Lodin et al. 2017).

We encountered many uncertainties when assessing the implications of converting pine sites to spruce stands. For example, few of the biodiversity assessments reviewed directly quantify the stand or landscape implications for specific taxonomic groups from such conversions. Similarly, the comparison of production outcomes was curtailed by the limited number of relevant studies available, their variability in methodology, and the specific suite of silvicultural prescriptions employed (e.g. soil preparation, planting density, thinning regimes). Likewise, as the practice of establishing spruce on sites traditionally used for pine is a relatively recent phenomenon, evidence of long-term implications is missing. Uncertainty also stems from how best to manage the identified risks that may develop from such conversions. For example, whereas the risk of storm damage in spruce stands can be mitigated by shortening the rotation and altering thinning practices (Valinger and Fridman 2011), reduced rotation lengths have their own suite of negative implications for biodiversity and some ecosystem services (Roberge et al. 2016; Felton et al. 2017). Alternatively, forest owners may benefit from establishing mixed stands (Holmström et al. 2018). This strategy is associated with a range of benefits to biodiversity and ecosystem services (Felton et al. 2016b), provides some insurance against the uncertainties of determining whether a site is best suited for spruce or pine, and can allow for some degree of browsing damage to pine without incurring significant losses to production. In either regard, it appears crucial that a better balance is achieved between the availability of suitable forage in the landscape, and the population abundance of large browsing herbivores (Wallgren et al. 2013).

## Conclusion

Global change and increasing pressure on natural resources provide both incentives and opportunities for people to change their choice of production of tree species. In the circumstances we assessed, such a change in tree species was linked to negative outcomes for biodiversity and many ecosystem services. The ongoing conversion of pine sites to spruce is essentially a large-scale uncontrolled experiment, for which every additional stand converted effectively locks in the extent of area susceptible to the risks, uncertainties, and potential adverse implications identified. A key concern is that unless mitigation efforts rapidly end these conversions, browsing pressure will intensify in remaining vulnerable stands, further reinforcing the expanded use of spruce. We, therefore, hope our findings clarify for forest owners, forest managers, and policy-makers the many adverse biodiversity and ecosystem service implications that can be expected if current efforts to stop stand conversions prove insufficient. In contrast, win-wins seem likely if measures to reverse ongoing losses of pine stands are successful.
